# Supplementary material for: Structure-based inhibitors of amyloid beta core suggest a common interface with tau
Source: eLife. 2019 Oct 15;8:e46924. doi: 10.7554/eLife.46924 (PMC6850776; doi:10.7554/eLife.46924)
Supplement: Source data 1. [file elife-46924-data1.docx]

**Supplemental Document, Extended ANOVA calculations**

**Figure 2A**

| Dunnett's multiple comparisons test | Mean Diff. | 95.00% CI of diff. | Significant? | Summary | Adjusted P Value |
| --- | --- | --- | --- | --- | --- |
|  |  |  |  |  |  |
| Aβ 1μM vs. +10μM L1 | -8.458 | -19.16 to 2.239 | No | ns | 0.1485 |
| Aβ 1μM vs. +10μM L2 | -0.6869 | -11.38 to 10.01 | No | ns | 0.9997 |
| Aβ 1μM vs. +10μM L3 | -7.283 | -17.98 to 3.415 | No | ns | 0.2505 |
| Aβ 1μM vs. +10μM L4 | -2.192 | -12.89 to 8.506 | No | ns | 0.9761 |
| Aβ 1μM vs. +10μM D1 | -39.79 | -50.49 to -29.09 | Yes | **** | 0.0001 |
| Aβ 1μM vs. +10μM D2 | -9.407 | -20.1 to 1.291 | No | ns | 0.0947 |

**Figure 2E**

| Dunnett's multiple comparisons test | Mean Diff. | 95.00% CI of diff. | Significant? | Summary | Adjusted P Value |
| --- | --- | --- | --- | --- | --- |
|  |  |  |  |  |  |
| Aβ 1μM vs. +10μM D1 | -54.87 | -64.09 to -45.65 | Yes | **** | 0.0001 |
| Aβ 1μM vs. +1μM D1 | -38.33 | -47.56 to -29.11 | Yes | **** | 0.0001 |
| Aβ 1μM vs. +200nM D1 | -22.21 | -31.43 to -12.99 | Yes | **** | 0.0001 |
| Aβ 1μM vs. +100nM D1 | -0.4758 | -9.697 to 8.746 | No | ns | 0.9998 |
| Aβ 1μM vs. +10μM D1b | -61.71 | -70.93 to -52.48 | Yes | **** | 0.0001 |
| Aβ 1μM vs. +1μM D1b | -35.25 | -44.47 to -26.03 | Yes | **** | 0.0001 |
| Aβ 1μM vs. +200nM D1b | -7.198 | -16.42 to 2.024 | No | ns | 0.2077 |
| Aβ 1μM vs. +100nM D1b | -0.05417 | -9.276 to 9.167 | No | ns | 0.9999 |
| Aβ 1μM vs. +10μM D1d | -65.05 | -74.27 to -55.83 | Yes | **** | 0.0001 |
| Aβ 1μM vs. +1μM D1d | -42.73 | -51.95 to -33.51 | Yes | **** | 0.0001 |
| Aβ 1μM vs. +200nM D1d | -13.64 | -22.86 to -4.415 | Yes | ** | 0.0010 |
| Aβ 1μM vs. +100nM D1d | -0.9327 | -10.15 to 8.289 | No | ns | 0.9996 |
| Aβ 1μM vs. +10μM LC | 5.962 | -3.26 to 15.18 | No | ns | 0.4173 |
| Aβ 1μM vs. +1μM LC | 1.271 | -7.951 to 10.49 | No | ns | 0.9994 |
| Aβ 1μM vs. +5μM D1 | -46.25 | -55.48 to -37.03 | Yes | **** | 0.0001 |
| Aβ 1μM vs. +5μM D1b | -49.96 | -59.18 to -40.74 | Yes | **** | 0.0001 |
| Aβ 1μM vs. +5μM D1d | -62.13 | -71.35 to -52.9 | Yes | **** | 0.0001 |

**Figure 2- Supplement 1A**

| Dunnett's multiple comparisons test | Mean Diff. | 95.00% CI of diff. | Significant? | Summary | Adjusted P Value |
| --- | --- | --- | --- | --- | --- |
|  |  |  |  |  |  |
| DPBS vs. L1 | -6.709 | -32.74 to 19.32 | No | ns | 0.9354 |
| DPBS vs. L2 | 0.4885 | -25.54 to 26.52 | No | ns | 0.9999 |
| DPBS vs. L3 | -14.84 | -40.87 to 11.19 | No | ns | 0.4026 |
| DPBS vs. L4 | -5.247 | -31.28 to 20.78 | No | ns | 0.9779 |
| DPBS vs. D1 | 1.03 | -25 to 27.06 | No | ns | 0.9999 |
| DPBS vs. D2 | 2.553 | -23.48 to 28.58 | No | ns | 0.9996 |

**Figure 2- Supplement 1B**

| Dunnett's multiple comparisons test | Mean Diff. | 95.00% CI of diff. | Significant? | Summary | Adjusted P Value |
| --- | --- | --- | --- | --- | --- |
|  |  |  |  |  |  |
| DPBS vs. D1 | -6.837 | -33.66 to 19.98 | No | ns | 0.9774 |
| DPBS vs. D1a | 8.399 | -18.42 to 35.22 | No | ns | 0.9311 |
| DPBS vs. D1b | 10.72 | -16.1 to 37.54 | No | ns | 0.8034 |
| DPBS vs. D1c | 1.626 | -25.19 to 28.45 | No | ns | 0.9997 |
| DPBS vs. D1d | -3.328 | -30.15 to 23.49 | No | ns | 0.9995 |
| DPBS vs. D1e | 6.09 | -20.73 to 32.91 | No | ns | 0.9895 |
| DPBS vs. D1f | -5.905 | -32.72 to 20.92 | No | ns | 0.9910 |
| DPBS vs. DMSO | 98.94 | 72.12 to 125.8 | Yes | **** | 0.0001 |
| DPBS vs. LC | -13.46 | -40.28 to 13.36 | No | ns | 0.6014 |

**Figure 2- Supplement 1C**

| Dunnett's multiple comparisons test | Mean Diff. | 95.00% CI of diff. | Significant? | Summary | Adjusted P Value |  |
| --- | --- | --- | --- | --- | --- | --- |
|  |  |  |  |  |  |  |
| Aβ 1μM vs. +1μM D1 | -35.88 | -54.59 to -17.17 | Yes | **** | 0.0001 |  |
| Aβ 1μM vs. +10μM D1 | -51.47 | -70.18 to -32.76 | Yes | **** | 0.0001 |  |
| Aβ 1μM vs. +1μM D1a | -27.92 | -46.63 to -9.215 | Yes | ** | 0.0012 |  |
| Aβ 1μM vs. +10μM D1a | -48.02 | -66.73 to -29.31 | Yes | **** | 0.0001 |  |
| Aβ 1μM vs. +1μM D1b | -42.02 | -60.72 to -23.31 | Yes | **** | 0.0001 |  |
| Aβ 1μM vs. +10μM D1b | -58.44 | -77.15 to -39.73 | Yes | **** | 0.0001 |  |
| Aβ 1μM vs. +1μM D1c | -11.06 | -29.76 to 7.649 | No | ns | 0.4971 |  |
| Aβ 1μM vs. +10μM D1c | -52.72 | -71.42 to -34.01 | Yes | **** | 0.0001 |  |
| Aβ 1μM vs. +1μM D1d | -46.88 | -65.58 to -28.17 | Yes | **** | 0.0001 |  |
| Aβ 1μM vs. +10μM D1d | -44.86 | -63.56 to -26.15 | Yes | **** | 0.0001 |  |
| Aβ 1μM vs. +1μM D1e | -36.71 | -55.42 to -18 | Yes | **** | 0.0001 |  |
| Aβ 1μM vs. +10μM D1e | -50.05 | -68.76 to -31.35 | Yes | **** | 0.0001 |  |
| Aβ 1μM vs. +1μM D1f | -38.33 | -57.04 to -19.62 | Yes | **** | 0.0001 |  |
| Aβ 1μM vs. +10μM D1f | -57.07 | -75.78 to -38.37 | Yes | **** | 0.0001 |  |
|  |  |  |  |  |  | |

**Figure 4A**

| Dunnett's multiple comparisons test | Mean Diff. | 95.00% CI of diff. | Significant? | Summary | Adjusted P Value |
| --- | --- | --- | --- | --- | --- |
|  |  |  |  |  |  |
| Aβ 1uM vs. +10μM D1 | -18.88 | -28.63 to -9.123 | Yes | **** | 0.0001 |
| Aβ 1uM vs. +5μM D1 | -9.123 | -18.88 to 0.6294 | No | ns | 0.0776 |
| Aβ 1uM vs. +1μM D1 | 0.5476 | -9.205 to 10.3 | No | ns | 0.9998 |
| Aβ 1uM vs. +500nM D1 | 6.573 | -3.18 to 16.33 | No | ns | 0.3539 |
| Aβ 1uM vs. +10μM D1b | -62.31 | -72.06 to -52.55 | Yes | **** | 0.0001 |
| Aβ 1uM vs. +5μM D1b | -15.69 | -25.45 to -5.942 | Yes | *** | 0.0004 |
| Aβ 1uM vs. +1μM D1b | 0.2508 | -9.502 to 10 | No | ns | 0.9999 |
| Aβ 1uM vs. +500nM D1b | 6.439 | -3.314 to 16.19 | No | ns | 0.3780 |
| Aβ 1uM vs. +10μM D1d | -62.81 | -72.57 to -53.06 | Yes | **** | 0.0001 |
| Aβ 1uM vs. +5μM D1d | -51.73 | -61.49 to -41.98 | Yes | **** | 0.0001 |
| Aβ 1uM vs. +1μM D1d | -39.53 | -49.28 to -29.78 | Yes | **** | 0.0001 |
| Aβ 1uM vs. +500nM D1d | -2.013 | -11.77 to 7.739 | No | ns | 0.9991 |
| Aβ 1uM vs. +10μM LC | -1.913 | -11.67 to 7.839 | No | ns | 0.9991 |
| Aβ 1uM vs. +1μM LC | -3.101 | -12.85 to 6.652 | No | ns | 0.9777 |
| Aβ 1uM vs. SDS | 34.54 | 24.79 to 44.29 | Yes | **** | 0.0001 |

**Figure 5D**

| Dunnett's multiple comparisons test | Mean Diff. | 95.00% CI of diff. | Significant? | Summary | Adjusted P Value |
| --- | --- | --- | --- | --- | --- |
|  |  |  |  |  |  |
| Aβ seeds vs. +250nM D1 | 55.36 | -937.1 to 1048 | No | ns | 0.9998 |
| Aβ seeds vs. +1μM D1 | -680 | -1672 to 312.5 | No | ns | 0.3809 |
| Aβ seeds vs. +5μM D1 | -1964 | -2957 to -971.9 | Yes | **** | 0.0001 |
| Aβ seeds vs. +20μM D1 | 1405 | 412 to 2397 | Yes | ** | 0.0012 |
| Aβ seeds vs. +250nM D1b | 348.2 | -644.3 to 1341 | No | ns | 0.9854 |
| Aβ seeds vs. +1μM D1b | 1108 | 115.4 to 2100 | Yes | * | 0.0196 |
| Aβ seeds vs. +5μM D1b | 1454 | 461.6 to 2447 | Yes | *** | 0.0008 |
| Aβ seeds vs. +20μM D1b | 1423 | 430.6 to 2416 | Yes | ** | 0.0011 |
| Aβ seeds vs. +250nM D1d | -1378 | -2371 to -385.9 | Yes | ** | 0.0016 |
| Aβ seeds vs. +1μM D1d | -1577 | -2569 to -584.3 | Yes | *** | 0.0002 |
| Aβ seeds vs. +5μM D1d | -1950 | -2943 to -957.6 | Yes | **** | 0.0001 |
| Aβ seeds vs. +20μM D1d | 1459 | 466.3 to 2451 | Yes | *** | 0.0008 |
| Aβ seeds vs. +5μM LC | -1180 | -2172 to -187.1 | Yes | * | 0.0105 |
| Aβ seeds vs. +20μM LC | -1812 | -2805 to -819.9 | Yes | **** | 0.0001 |
| Aβ seeds vs. Vehicle | 1532 | 539.4 to 2524 | Yes | *** | 0.0004 |
|  |  |  |  |  |  |
|  |  |  |  |  |  |

**Figure 5- Supplement 1C**

| Tukey's multiple comparisons test | Mean Diff. | 95.00% CI of diff. | Significant? | Summary | Adjusted P Value |
| --- | --- | --- | --- | --- | --- |
|  |  |  |  |  |  |
| hIAPP vs. Tau k18+ | -107083 | -119588 to -94578 | Yes | **** | <0.0001 |
| hIAPP vs. TDP43 | 8.117 | -12497 to 12513 | No | ns | >0.9999 |
| hIAPP vs. α-syn | -75.33 | -12580 to 12430 | No | ns | >0.9999 |
| hIAPP vs. TTR | 11.72 | -12493 to 12517 | No | ns | >0.9999 |
| hIAPP vs. Vehicle | 0.3929 | -12505 to 12505 | No | ns | >0.9999 |
| Tau k18+ vs. TDP43 | 107091 | 94586 to 119596 | Yes | **** | <0.0001 |
| Tau k18+ vs. α-syn | 107007 | 94502 to 119512 | Yes | **** | <0.0001 |
| Tau k18+ vs. TTR | 107094 | 94589 to 119599 | Yes | **** | <0.0001 |
| Tau k18+ vs. Vehicle | 107083 | 94578 to 119588 | Yes | **** | <0.0001 |
| TDP43 vs. α-syn | -83.44 | -12588 to 12422 | No | ns | >0.9999 |
| TDP43 vs. TTR | 3.606 | -12501 to 12509 | No | ns | >0.9999 |
| TDP43 vs. Vehicle | -7.725 | -12513 to 12497 | No | ns | >0.9999 |
| α-syn vs. TTR | 87.05 | -12418 to 12592 | No | ns | >0.9999 |
| α-syn vs. Vehicle | 75.72 | -12429 to 12581 | No | ns | >0.9999 |
| TTR vs. Vehicle | -11.33 | -12516 to 12494 | No | ns | >0.9999 |

**Figure 6B**

| Dunnett's multiple comparisons test | Mean Diff. | 95.00% CI of diff. | Significant? | Summary | Adjusted P Value |
| --- | --- | --- | --- | --- | --- |
|  |  |  |  |  |  |
| No Inhibitor vs. +250nM D1 | -3996 | -20949 to 12957 | No | ns | 0.9989 |
| No Inhibitor vs. +1μM D1 | 2016 | -14937 to 18969 | No | ns | 0.9995 |
| No Inhibitor vs. +5μM D1 | 3788 | -13165 to 20741 | No | ns | 0.9990 |
| No Inhibitor vs. +20μM D1 | -101.9 | -17055 to 16851 | No | ns | 0.9999 |
| No Inhibitor vs. +50μM D1 | 25346 | 8393 to 42299 | Yes | *** | 0.0007 |
| No Inhibitor vs. +75μM D1 | 30959 | 14006 to 47912 | Yes | **** | 0.0001 |
| No Inhibitor vs. +250nM D1b | -13577 | -30530 to 3376 | No | ns | 0.1917 |
| No Inhibitor vs. +1μM D1b | -27824 | -44777 to -10871 | Yes | *** | 0.0002 |
| No Inhibitor vs. +5μM D1b | 23176 | 6223 to 40129 | Yes | ** | 0.0023 |
| No Inhibitor vs. +20μM D1b | 38844 | 21891 to 55797 | Yes | **** | 0.0001 |
| No Inhibitor vs. +50μM D1b | 39679 | 22726 to 56632 | Yes | **** | 0.0001 |
| No Inhibitor vs. +75μM D1b | 42828 | 25875 to 59781 | Yes | **** | 0.0001 |
| No Inhibitor vs. +250nM D1d | -6354 | -23307 to 10599 | No | ns | 0.9602 |
| No Inhibitor vs. +1μM D1d | -14914 | -31867 to 2039 | No | ns | 0.1164 |
| No Inhibitor vs. +5μM D1d | 8425 | -8528 to 25378 | No | ns | 0.7711 |
| No Inhibitor vs. +20μM D1d | -1682 | -18635 to 15271 | No | ns | 0.9996 |
| No Inhibitor vs. +50μM D1d | -12752 | -29704 to 4201 | No | ns | 0.2547 |
| No Inhibitor vs. +75μM D1d | 30684 | 13731 to 47637 | Yes | **** | 0.0001 |
| No Inhibitor vs. +5μM LC | 7794 | -9158 to 24747 | No | ns | 0.8450 |
| No Inhibitor vs. +75μM LC | -18642 | -35595 to -1689 | Yes | * | 0.0232 |
| No Inhibitor vs. vehicle | 40225 | 23272 to 57178 | Yes | **** | 0.0001 |

**Figure 6D**

**Mutant 1**

| Dunnett's multiple comparisons test | Mean Diff. | 95.00% CI of diff. | Significant? | Summary | Adjusted P Value |
| --- | --- | --- | --- | --- | --- |
|  |  |  |  |  |  |
| no inhibitor vs. +250nM D1b | 5690 | -9089 to 20468 | No | ns | 0.8060 |
| no inhibitor vs. +1μM D1b | 14122 | -657 to 28900 | No | ns | 0.0648 |
| no inhibitor vs. +5μM D1b | 34845 | 20066 to 49623 | Yes | **** | 0.0001 |
| no inhibitor vs. +20μM D1b | 43038 | 28259 to 57816 | Yes | **** | 0.0001 |
| no inhibitor vs. +50μM D1b | 39149 | 24370 to 53928 | Yes | **** | 0.0001 |
| no inhibitor vs. +75μM D1d | 39724 | 24946 to 54503 | Yes | **** | 0.0001 |

**Mutant 5**

| Dunnett's multiple comparisons test | Mean Diff. | 95.00% CI of diff. | Significant? | Summary | Adjusted P Value |
| --- | --- | --- | --- | --- | --- |
|  |  |  |  |  |  |
| no inhibitor vs. +250nM D1b | -1985 | -3193 to 3099 | No | ns | 0.7961 |
| no inhibitor vs. +1μM D1b | -3125 | -4333 to -1918 | No | ns | 0.3731 |
| no inhibitor vs. +5μM D1b | 8168 | 6961 to 9375 | Yes | **** | 0.0001 |
| no inhibitor vs. +20μM D1b | 8628 | 7421 to 9835 | Yes | **** | 0.0001 |
| no inhibitor vs. +50μM D1b | 8517 | 7310 to 9725 | Yes | **** | 0.0001 |
| no inhibitor vs. +75μM D1d | 8286 | 7078 to 9493 | Yes | **** | 0.0001 |
| **3R tau**  Dunnett's multiple comparisons test | Mean Diff. | 95.00% CI of diff. | Significant? | Summary | Adjusted P Value |
|  |  |  |  |  |  |
| no inhibitor vs. +250nM D1b | 2668 | -2592 to 7928 | No | ns | 0.6469 |
| no inhibitor vs. +1μM D1b | -1237 | -6497 to 4023 | No | ns | 0.9925 |
| no inhibitor vs. +5μM D1b | 10097 | 4837 to 15357 | Yes | **** | 0.0001 |
| no inhibitor vs. +20μM D1b | 12597 | 7337 to 17856 | Yes | **** | 0.0001 |
| no inhibitor vs. +50μM D1b | 12294 | 7034 to 17554 | Yes | **** | 0.0001 |
| no inhibitor vs. +75μM D1d | 11812 | 6552 to 17071 | Yes | **** | 0.0001 |

**Figure 6- Supplement 1D**

**Mutant 2**

| Dunnett's multiple comparisons test | Mean Diff. | 95.00% CI of diff. | Significant? | Summary | Adjusted P Value |
| --- | --- | --- | --- | --- | --- |
|  |  |  |  |  |  |
| no inhibitor vs. +250nM D1b | -603 | -4058 to 2852 | No | ns | 0.9954 |
| no inhibitor vs. +1μM D1b | -4087 | -7542 to -631.8 | Yes | * | 0.0165 |
| no inhibitor vs. +5μM D1b | -2439 | -5894 to 1016 | No | ns | 0.2461 |
| no inhibitor vs. +20μM D1b | 892.5 | -2563 to 4348 | No | ns | 0.9665 |
| no inhibitor vs. +50μM D1b | 3711 | 255.9 to 7166 | Yes | * | 0.0322 |
| no inhibitor vs. +75μM D1d | 3319 | -136 to 6774 | No | ns | 0.0629 |
| **Mutant 3**  Dunnett's multiple comparisons test | Mean Diff. | 95.00% CI of diff. | Significant? | Summary | Adjusted P Value |
|  |  |  |  |  |  |
| no inhibitor vs. +250nM D1b | 3354 | -16944 to 23651 | No | ns | 0.9971 |
| no inhibitor vs. +1μM D1b | 4388 | -15909 to 24685 | No | ns | 0.9880 |
| no inhibitor vs. +5μM D1b | 6491 | -13807 to 26788 | No | ns | 0.9067 |
| no inhibitor vs. +20μM D1b | 5557 | -14741 to 25854 | No | ns | 0.9548 |
| no inhibitor vs. +50μM D1b | 7049 | -13248 to 27346 | No | ns | 0.8683 |
| no inhibitor vs. +75μM D1d | 2768 | -17530 to 23065 | No | ns | 0.9994 |
| **Mutant 4**  Dunnett's multiple comparisons test | Mean Diff. | 95.00% CI of diff. | Significant? | Summary | Adjusted P Value |
|  |  |  |  |  |  |
| no inhibitor vs. +250nM D1b | -25176 | -35357 to -14995 | Yes | **** | 0.0001 |
| no inhibitor vs. +1μM D1b | -17608 | -27789 to -7427 | Yes | *** | 0.0009 |
| no inhibitor vs. +5μM D1b | 2839 | -7342 to 13020 | No | ns | 0.9120 |
| no inhibitor vs. +20μM D1b | 9980 | -200.6 to 20161 | No | ns | 0.0556 |
| no inhibitor vs. +50μM D1b | 30470 | 20289 to 40651 | Yes | **** | 0.0001 |
| no inhibitor vs. +75μM D1d | 36116 | 25935 to 46297 | Yes | **** | 0.0001 |

**Figure 6- Supplement 1E**

| Tukey's multiple comparisons test | Mean Diff. | 95.00% CI of diff. | Significant? | Summary | Adjusted P Value |
| --- | --- | --- | --- | --- | --- |
|  |  |  |  |  |  |
| WT vs. Null Mutant | 76855 | 63506 to 90204 | Yes | **** | <0.0001 |
| WT vs. Vehicle | 76868 | 63520 to 90217 | Yes | **** | <0.0001 |
| Null Mutant vs. Vehicle | 13.44 | -13335 to 13362 | No | ns | >0.9999 |
|  |  |  |  |  |  |

**Figure 6- Supplement 1F**

| Dunnett's multiple comparisons test | Mean Diff. | | 95.00% CI of diff. | Significant? | Summary | Adjusted P Value |
| --- | --- | --- | --- | --- | --- | --- |
| **Mutant 1**  no inhibitor vs. +5μM LC | -2957 | | -29795 to 23882 | No | ns | 0.9983 |
| no inhibitor vs. +20μM LC | 7240 | | -19598 to 34079 | No | ns | 0.9226 |
| no inhibitor vs. +50μM LC | 18375 | | -8464 to 45213 | No | ns | 0.2461 |
| **Mutant 2**  no inhibitor vs. +5μM LC | | -3419 | -10102 to 3264 | No | ns | 0.5027 |
| no inhibitor vs. +20μM LC | | -3657 | -10340 to 3026 | No | ns | 0.4404 |
| no inhibitor vs. +50μM LC | | -7308 | -13991 to -624.7 | Yes | * | 0.0301 |
| **Mutant 3**  no inhibitor vs. +5μM LC | -23210 | | -71133 to 24714 | No | ns | 0.5532 |
| no inhibitor vs. +20μM LC | -20411 | | -68335 to 27513 | No | ns | 0.6653 |
| no inhibitor vs. +50μM LC | -18052 | | -65976 to 29872 | No | ns | 0.7587 |
| **Mutant 4**  no inhibitor vs. +5μM LC | -26690 | | -37333 to -16047 | Yes | **** | 0.0001 |
| no inhibitor vs. +20μM LC | -21216 | | -31859 to -10573 | Yes | *** | 0.0003 |
| no inhibitor vs. +50μM LC | -2440 | | -13083 to 8204 | No | ns | 0.9606 |
| **Mutant 5**  no inhibitor vs. +5μM LC | -20130 | | -30726 to -9535 | Yes | *** | 0.0004 |
| no inhibitor vs. +20μM LC | -19657 | | -30253 to -9062 | Yes | *** | 0.0005 |
| no inhibitor vs. +50μM LC | -24327 | | -34923 to -13732 | Yes | **** | 0.0001 |
| **3R tau**  no inhibitor vs. +5μM LC | 2687 | | -2516 to 7891 | No | ns | 0.4939 |
| no inhibitor vs. +20μM LC | 1540 | | -3663 to 6743 | No | ns | 0.8901 |
| no inhibitor vs. +50μM LC | 1424 | | -3780 to 6627 | No | ns | 0.9183 |

**Figure 7**

| Dunnett's multiple comparisons test | Mean Diff. | 95.00% CI of diff. | Significant? | Summary | Adjusted P Value |
| --- | --- | --- | --- | --- | --- |
|  |  |  |  |  |  |
| **AD- Hippocampus**  Seed vs. +D1 | 15805 | 2618 to 28992 | Yes | * | 0.0196 |
| Seed vs. +D1b | 28818 | 15630 to 42005 | Yes | *** | 0.0003 |
| Seed vs. +D1d | -14039 | -27227 to -852.2 | Yes | * | 0.0368 |
| Seed vs. +LC | 4458 | -8729 to 17646 | No | ns | 0.7330 |
| **AD- Frontal Lobe**  Seed vs. +D1 | 5706 | 1186 to 10226 | Yes | * | 0.0145 |
| Seed vs. +D1b | 11605 | 7084 to 16125 | Yes | **** | 0.0001 |
| Seed vs. +D1d | -5416 | -9936 to -895.7 | Yes | * | 0.0196 |
| Seed vs. +LC | -2236 | -6756 to 2284 | No | ns | 0.4530 |
| **AD- Occipital Lobe**  Seed vs. +D1 | 1275 | -922.8 to 3472 | No | ns | 0.3278 |
| Seed vs. +D1b | 7159 | 4962 to 9357 | Yes | **** | 0.0001 |
| Seed vs. +D1d | 4656 | 2458 to 6853 | Yes | *** | 0.0004 |
| Seed vs. +LC | 2617 | 419.3 to 4814 | Yes | * | 0.0203 |
| **PSP**  Seed vs. +D1 | 4264 | 2317 to 6212 | Yes | *** | 0.0003 |
| Seed vs. +D1b | 7289 | 5341 to 9236 | Yes | **** | 0.0001 |
| Seed vs. +D1d | 3850 | 1902 to 5797 | Yes | *** | 0.0007 |
| Seed vs. +LC | 1017 | -930.5 to 2964 | No | ns | 0.4097 |
