# Supplementary material for: Structure-based inhibitors of amyloid beta core suggest a common interface with tau
Source: eLife. 2019 Oct 15;8:e46924. doi: 10.7554/eLife.46924 (PMC6850776; doi:10.7554/eLife.46924)
Supplement: Supplementary file 1. [file elife-46924-supp1.docx]

**Supplemental Table 1. Computed binding properties of designed inhibitors to amyloid-beta _16_KLVFFAEN_23_**

| Peptide  Name | Sequence | MW (g/mol) | Hydropathicity (GRAVY) | Binding Energy  (R.E.U.) | SA Buried  (Å^2^) | Shape Complementarity |
| --- | --- | --- | --- | --- | --- | --- |
| L1 | KWYFIE | 885 | -0.38 | -4.41 | 760 | 0.76 |
| L2 | NLYVRE | 793 | -0.80 | -5.02 | 761 | 0.73 |
| L3 | ERLYHFME | 1124 | -0.94 | -4.69 | 1016 | 0.79 |
| L4 | QRVYRTWQ | 1136 | -1.84 | -4.75 | 1026 | 0.75 |
| LC | LYIWVQ | 821 | 1.13 | -4.25 | 719 | 0.75 |
| D1 | D-LYIWVQ | 821 | 1.13 | -4.92 | 771 | 0.70 |
| D1a | D-LYIWIQMQ | 1094 | 0.69 | -5.33 | 1149 | 0.71 |
| D1b | D-LYIWIWRT | 1150 | 0.56 | -6.11 | 1235 | 0.72 |
| D1c | D-LYIWIWFS | 1127 | 1.46 | -5.95 | 1215 | 0.71 |
| D1d | D-LYIWIQKT | 1064 | 0.31 | -5.25 | 1140 | 0.70 |
| D1e | D-MYIWVQ | 839 | 0.82 | -4.94 | 776 | 0.70 |
| D1f | D-MYIWRQ | 896 | -0.63 | -4.40 | 802 | 0.70 |
| D2 | D-MLIVRN | 745 | 1.07 | -4.17 | 760 | 0.70 |
